# Successful treatment of refractory focal eosinophilic myositis with extra-ocular muscle involvement using tocilizumab: a case report

**DOI:** 10.3389/fimmu.2026.1850543

**Published:** 2026-05-25

**Authors:** Haijun Liu, Qianhua Li, Xia Ouyang, Shuyu Chen, Henglun Liang, Yufeng Ye, Huiyuan Cao, Yong Xue, Lie Dai

**Affiliations:** 1 The Affiliated Panyu Central Hospital of Guangzhou Medical University, Guangzhou, China; 2 Sun Yat-Sen Memorial Hospital, Sun Yat-Sen University, Guangzhou, China

**Keywords:** eosinophilic myositis, focal, ocular myositis, tocilizumab, case report

## Abstract

Eosinophilic myositis (EM) is a rare subset of idiopathic inflammatory myopathies characterized by prominent eosinophilic infiltration of skeletal muscle. It encompasses focal eosinophilic myositis (FEM), diffuse eosinophilic myositis, and eosinophilic perimyositis. FEM typically presents with single or multiple lesions, most commonly involving the limbs. However, involvement of extra-ocular muscles in FEM is exceedingly rare and has not been well documented. Here, we describe a 65-year-old woman who initially presented with chest wall pain. Magnetic resonance imaging (MRI) and muscle biopsy confirmed multifocal FEM. Notably, the patient also exhibited concomitant involvement of extra-ocular muscles. Treatment with prednisone combined with conventional immunosuppressants led to marked regression of truncal lesions. However, during glucocorticoid tapering, the patient experienced a relapse of myositis confined to the left extra-ocular muscles. She relapsed with left eyelid swelling, tearing, binocular diplopia, exotropia, and impaired adduction of the left eye. This ocular relapse was refractory to glucocorticoids and multiple immunosuppressive agents. Tocilizumab, administered at 8 mg/kg intravenously every 4 weeks, was associated with gradual improvement in diplopia and ocular motility, reduction in extraocular muscle swelling on MRI, and sustained clinical benefit during follow-up. These findings suggest that extra-ocular muscle involvement in FEM may define a more treatment-resistant phenotype, and tocilizumab may represent a promising therapeutic option in this context.

## Introduction

Eosinophilic myositis (EM) is a rare subgroup of idiopathic inflammatory myopathies distinguished by prominent eosinophilic infiltration of skeletal muscle (1). EM is classically categorized into focal eosinophilic myositis (FEM), diffuse eosinophilic myositis, and eosinophilic perimyositis based on the pattern and extent of muscle involvement (2). Among these, FEM is characterized by localized muscle inflammation, most frequently affecting the limbs, and is generally considered to have a favorable prognosis with appropriate immunosuppressive therapy (3).

Extra-ocular muscle involvement in myositis is most commonly associated with idiopathic orbital myositis, a distinct entity often presenting with acute orbital pain, diplopia, and unilateral extra-ocular muscle enlargement (4, 5). In contrast, involvement of extra-ocular muscles in the context of FEM is exceptionally rare, with no well-documented cases reported in the literature to date. Consequently, the clinical course, treatment response, and optimal management of such cases remain poorly defined.

We report a case of multifocal FEM that initially involved truncal muscles and subsequently relapsed with isolated extra-ocular muscle involvement following glucocorticoid tapering. The ocular relapse was refractory to multiple conventional immunosuppressive agents but ultimately responded to tocilizumab, an interleukin-6 (IL-6) receptor antagonist. This case represents a rare description of extra-ocular muscle involvement in FEM and the first use of tocilizumab for this indication, highlighting a potentially treatment-resistant phenotype and a novel therapeutic strategy.

## Case presentation

A 65-year-old previously healthy woman was admitted to the Thoracic Surgery Unit on April 3, 2023, for evaluation of a chest wall mass that had been present for three months. The patient initially experienced spontaneous right anterior chest pain. Computed tomography (CT) of the chest revealed soft tissue swelling surrounding the right 5th and 6th costal arches. Outpatient therapy with diclofenac temporarily alleviated the pain and swelling. However, two weeks prior to admission, the right chest wall became swollen and painful again. Ultrasound imaging demonstrated thickening of the subcutaneous muscle layers with increased vascularity.

Upon admission, laboratory studies revealed: white blood cells 9.43 × 10^9^/L, hemoglobin 117 g/L, platelets 446 × 10^9^/L, eosinophils 0.23 × 10^9^/L (reference range, 0.02-0.5 × 10^9^/L); urinalysis showed occult blood (+) but no protein. No ova or parasites were detected on microscopic examination of stool samples. Other relevant results included: alanine aminotransferase 9 U/L, creatinine 69 μmol/L, albumin 32.6 g/L, creatine kinase 76 U/L (reference range, 26–140 U/L), C-reactive protein (CRP) 53.53 mg/L (reference range, < 5 mg/L), erythrocyte sedimentation rate (ESR) 97 mm/h (reference range, < 20 mm/h), and procalcitonin 0.04 ng/mL. Tumor markers (CEA, CA-199, CA-724, CA-125, AFP) were within reference ranges. T-SPOT.TB and repeated sputum examinations for acid-fast bacilli were negative. Hepatitis B surface antigen, hepatitis C antibody, syphilis antibody, and HIV screening antibody were all negative. No abnormalities were detected on electrocardiogram, echocardiography, or lung CT.

**Figure 1:**
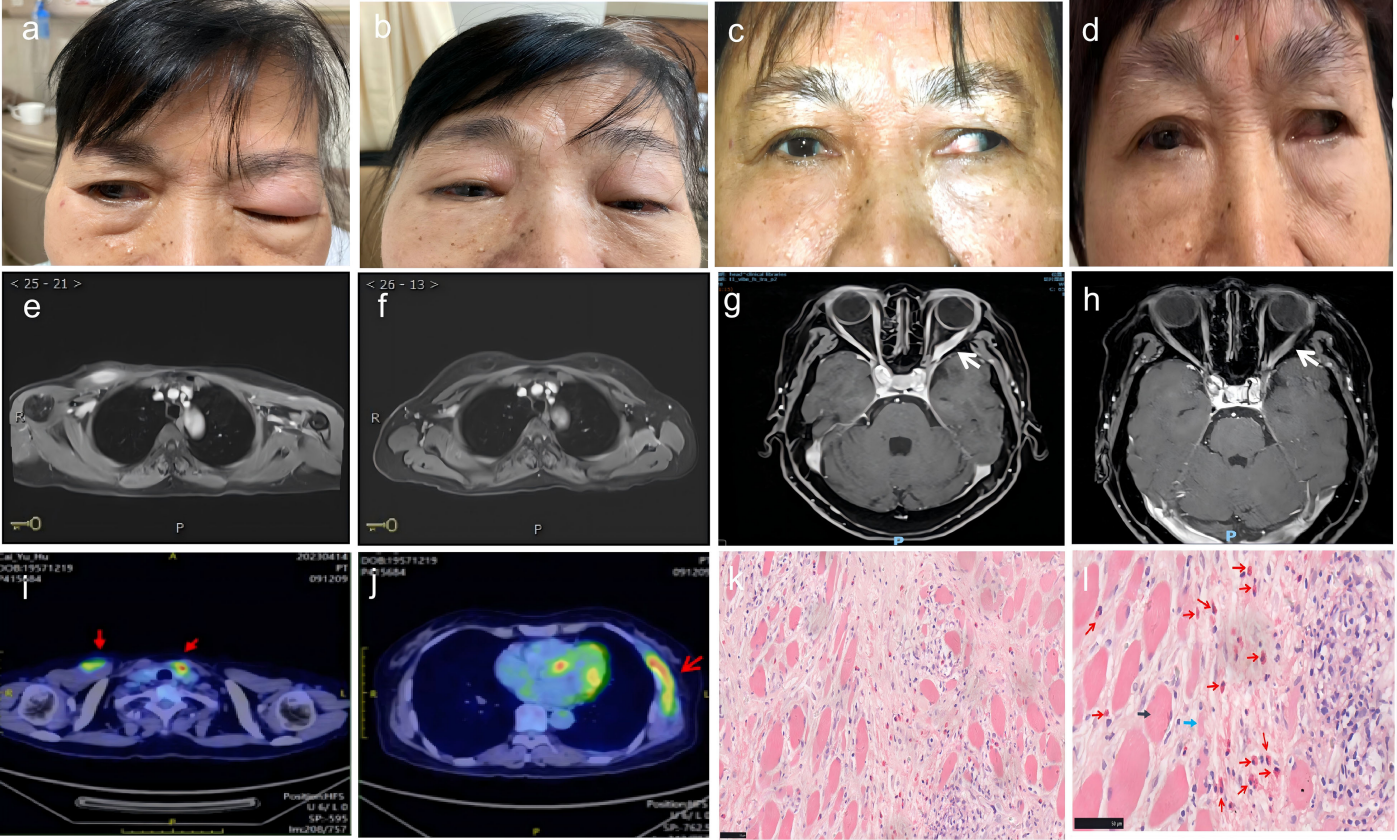
Clinical, radiological, PET-CT, and pathological findings. **(a)** Pre-treatment swelling of the left eyelid. **(b)** Reduction in left eyelid swelling after 1 week of glucocorticoid therapy. **(c)** Recurrence of left eyelid swelling presenting with impaired adduction and persistent abduction of the left eye. **(d)** Slight improvement in adduction amplitude of the left eye following tocilizumab treatment. **(e)** MRI of the chest wall showed pre-treatment localized myositis of the right pectoralis major muscle. **(f)** MRI of the chest wall showed significant resolution of localized myositis in the right pectoralis major muscle after 2 months of glucocorticoid therapy. **(g)** MRI of the left eye at recurrence showing swelling of the lateral rectus muscle. **(h)** Post-tocilizumab MRI demonstrating reduced swelling of the left lateral rectus muscle. i-j. PET-CT demonstrated diffuse and focal swelling with increased glucose metabolism in several muscles, including the right inferior pharyngeal constrictor muscle, right pectoralis major muscle, left sternothyroid muscle. **(k)** Pathological examination of a biopsy from the right pectoralis major muscle revealed focal small lesions with red staining suggesting necrosis, with extensive infiltration of lymphocytes, plasma cells, and eosinophils with a density of 56 per square millimeter (×100). **(l)** high-power views (×400) show eosinophils (red arrows) and muscle fibers (black arrow), possibly degenerated muscle fibers (blue arrow).

Contrast-enhanced magnetic resonance imaging (MRI) of the chest wall revealed multifocal muscle edema and abnormal enhancement involving the right pectoralis major and left serratus anterior, among other muscles (**Figure 1e**). During hospitalization, the patient developed worsening chest wall pain, swelling in the right anterior neck region, hoarseness, left eyelid edema, and mild dysphagia. Laboratory tests showed persistent elevation of ESR and CRP, with no evidence of malignancy, prompting suspicion of an autoimmune process. She was subsequently transferred to the Department of Rheumatology and Immunology for further evaluation.

On physical examination, left eyelid swelling (**Figure 1a**) and hoarseness were noted, but no significant tenderness was present over the chest wall, and muscle strength in all four limbs was normal. Additional laboratory tests were negative for antinuclear antibodies (ANA), anti-double-stranded DNA (ds-DNA), extractable nuclear antigen (ENA) antibodies, antineutrophil cytoplasmic antibodies (ANCA), rheumatoid factor (RF), and anti-cyclic citrullinated peptide (CCP) antibodies. ASO and anti-DNase B levels were normal. Tests for fungal infections, *Toxoplasma gondii* antibodies, herpes simplex virus antibodies, and cytomegalovirus antibodies were all negative. A myositis-specific autoantibody panel also returned negative results. Serum IgG4 level was 784.6 mg/L (reference range, < 2000mg/L), and IL-6 was 18.64ng/mL (reference range, 0-5.4pg/mL). Electromyography of the four limbs showed no abnormalities.

Positron emission tomography-computed tomography (PET-CT) demonstrated diffuse and focal swelling with increased glucose metabolism in several muscles (**Figures 1i, j**), including the right medial pterygoid, right pharyngeal constrictor, right longus capitis, right longus colli, right pectoralis major, left sternohyoid, left sternothyroid, left pectoralis minor, left serratus anterior, left intercostal, left teres major, and left latissimus dorsi. Left eyelid swelling was noted but without increased glucose uptake.

Pathological examination of a biopsy from the right pectoralis major muscle revealed focal eosinophilic necrosis-like changes under low-power magnification (×100), with surrounding fibrous tissue proliferation. No prominent vascular proliferation, granulomatous-like structures, or ganglion-like cell proliferation was observed. Under high-power magnification (×400), fibrous tissue proliferation between skeletal muscle fibers was noted, accompanied by focal infiltration of lymphocytes, plasma cells, and eosinophils, with a density of approximately 56 eosinophils per square millimeter. Partial atrophy of skeletal muscle fibers was also present (**Figures 1k, l**). No immunohistochemical staining was performed because the hematoxylin-eosin morphology was considered sufficient to demonstrate eosinophilic myositis and exclude malignancy in the sampled tissue. Based on the clinicopathological and imaging findings, a diagnosis of multifocal FEM was established.

On April 20, 2023, treatment was initiated with prednisone (50 mg daily for one month, then the dose was tapered by 5mg every two weeks to 10mg daily) and methotrexate (15 mg weekly), resulting in significant relief of chest pain and partial regression of eyelid swelling (**Figure 1b**) and resolution of hoarseness and dysphagia. MRI in May 2023 showed near-complete resolution of the right chest wall swelling (**Figure 1f**), allowing gradual tapering of glucocorticoids. However, by December 2023, when the prednisone dose was reduced to 10 mg daily (cumulative dose during the induction-and-taper phase, approximately was 4,580 mg), the patient developed recurrent left eyelid swelling and persistent left eye abduction with loss of adduction (**Figure 1c**) accompanied by left eye tearing and diplopia. High-dose glucocorticoids (Prednisone 50 mg daily for one month, then tapered by 5 mg per week to 10 mg daily) were reintroduced, leading to prompt resolution of eyelid edema and tearing; however, the abduction palsy persisted with diplopia persisted. Eosinophils counts and CK remained normal throughout.

**Figure 2:**
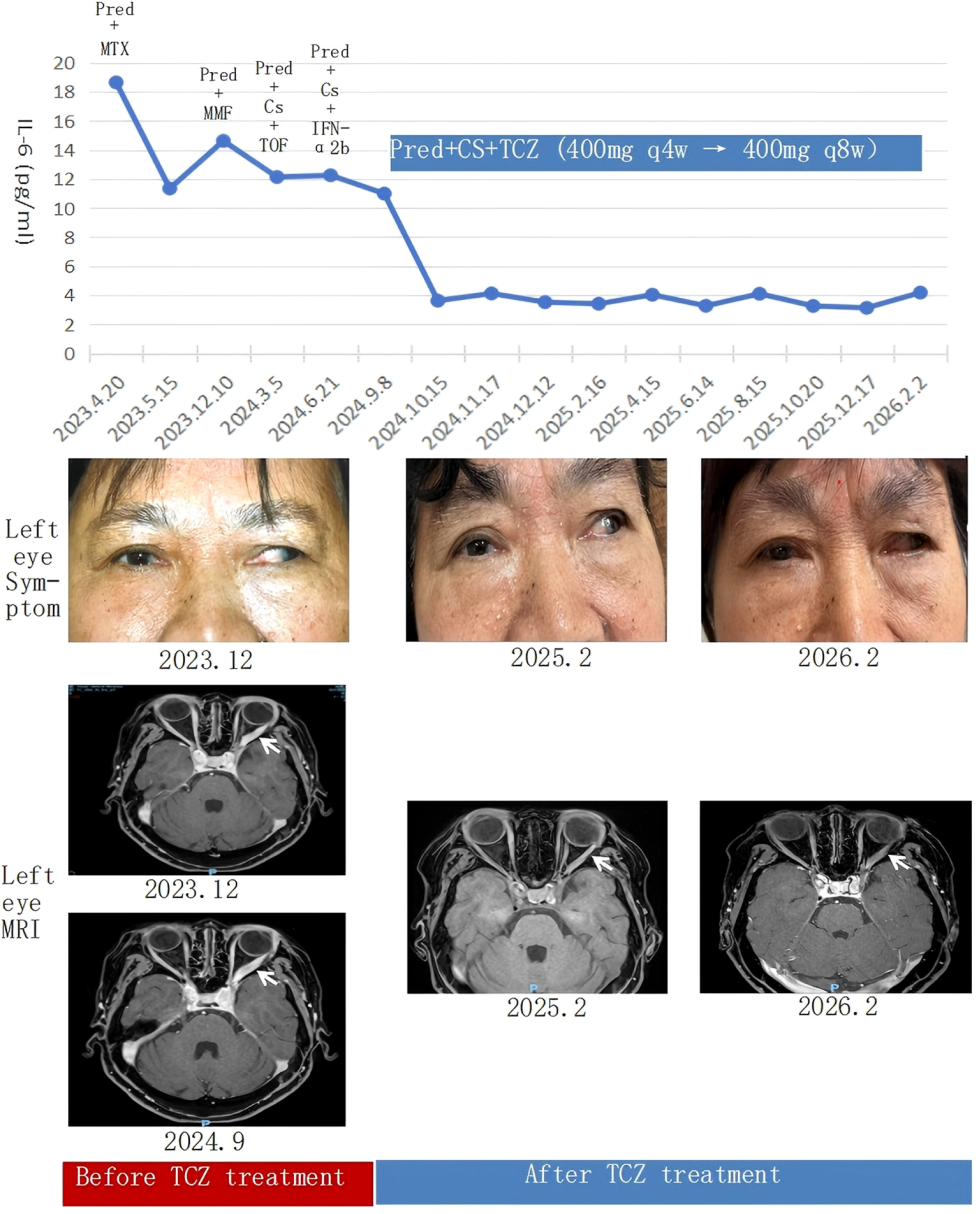
Graphical timeline of the treatment strategy, and outcomes. IL, Interleukin; Pred, Prednisone; MTX, Methotrexate; MMF, Mycophenolate Mofetil; Cs, Ciclosporin; TOF, Tofacitinib; IFN, Interferon, TCZ, Tocilizumab; MRI, Magnetic Resonance Imaging; q4w, every 4 weeks; q8w, every 8 weeks.

Orbital MRI revealed fusiform enlargement and T2-hyperintensity of the left extra-ocular muscles (**Figure 1g**). Given these findings were interpreted as disease relapse, methotrexate was switched to mycophenolate mofetil (0.75 g twice daily). By March 2024, there was no improvement in ocular motility. Consequently, mycophenolate mofetil was replaced by ciclosporin (75 mg twice daily), and tofacitinib (5 mg daily) was added. In June 2024, tofacitinib was discontinued and interferon-α2b was introduced, but after three months, no objective benefit was observed. In September 2024, the treatment regimen was revised to low-dose prednisone (10mg daily, cumulative prednisone dose by that time, approximately 10,460 mg), ciclosporin, and monthly tocilizumab (8mg/kg, body weight 51Kg, 400 mg intravenously). The IL-6 level before tocilizumab initiation was 10.99 pg/mL. During the first to second month after tocilizumab initiation, the patient reported no improvement in left eye exotropia, inability to adduct, or binocular diplopia. The IL-6 level decreased to 3.66 pg/mL (**Figure 2**). After three months of this combination therapy, the patient regained partial adduction of the left eye (**Figure 2**), with mild improvement in diplopia, and repeat orbital MRI demonstrated a significant reduction in extra-ocular muscle swelling (**Figure 1h**, **2**). The improvement in binocular diplopia reduced interference with daily activities, and both the patient and her family reported satisfaction with the clinical response. Tocilizumab was administered monthly for four months, after which the interval was extended to every two months for financial reasons. Left eye adduction and diplopia gradually continued to improve. At the latest follow-up in February 2026, the patient showed marked improvement in left eye adduction function (**Figure 1d**, **2**), and serum IL-6 was 4.18 pg/mL, CK and eosinophil counts remained normal, and orbital MRI showed sustained reduction in swelling of the left extraocular muscles (**Figure 2**). After tocilizumab therapy, she noticed gradual reduction in diplopia and improvement in the ability to move the left eye inward. She and her family expressed satisfaction with the clinical improvement and agreed to continue maintenance treatment with close follow-up.

## Discussion

We report a case of focal eosinophilic myositis (FEM) with two distinctive and previously undescribed features: involvement of extra-ocular muscles, and successful treatment with tocilizumab after failure of multiple conventional immunosuppressive agents. These observations carry important clinical implications for the recognition and management of this rare disease.

The patient presented with painful swelling of multiple skeletal muscles, elevated inflammatory markers (ESR and CRP), and imaging evidence of localized muscular enlargement. Muscle biopsy confirmed eosinophilic myositis, with extensive eosinophilic infiltration between muscle fascicles, with a density exceeding 5.79 per square millimeter, a hallmark feature of EM (3). The diagnosis of FEM was further supported by the multifocal but localized pattern of muscle involvement and the absence of diffuse myopathy or systemic features characteristic of other inflammatory myopathies (3).

The most notable aspect of this case is the involvement of extra-ocular muscles, which has rarely been previously reported in FEM. Extra-ocular muscle inflammation is most commonly encountered in idiopathic orbital myositis, a distinct condition often presenting with acute orbital pain, diplopia, and unilateral muscle enlargement that typically responds well to glucocorticoids (4, 5). In contrast, the ocular involvement in our patient occurred as a relapse of known FEM after glucocorticoid tapering, suggesting that this represented a manifestation of the same disease process rather than a separate entity. This raises the possibility that FEM may rarely extend to extra-ocular muscles, and when it does, it may herald a more treatment-resistant phenotype.

The patient’s ocular relapse proved refractory to a range of immunosuppressive therapies, including high-dose glucocorticoids, methotrexate, mycophenolate mofetil, ciclosporin, tofacitinib, and interferon-α2b. This pattern of treatment resistance is consistent with the known difficulty in managing orbital myositis, which is frequently associated with relapses and incomplete responses to conventional therapy (5, 6). The unique anatomical and immunological features of extra-ocular muscles—including the presence of the blood-ocular barrier, distinct muscle fiber types, and specialized immune microenvironment—may contribute to their relative insensitivity to systemic immunosuppression (7, 8).

The successful use of tocilizumab in this patient represents a novel therapeutic approach for refractory FEM with extra-ocular muscle involvement. Tocilizumab is a humanized monoclonal antibody targeting the IL-6 receptor, approved for the treatment of rheumatoid arthritis, giant cell arteritis, and other inflammatory conditions (9). IL-6 is a pleiotropic cytokine that plays complex roles in muscle physiology; chronically elevated IL-6 levels in myositis may impair muscle regeneration, promote fibrosis, and contribute to persistent inflammation (10, 11).A prospective open-label pilot study demonstrated that tocilizumab was effective in patients with refractory immune-mediated necrotizing myopathies, with 7 out of 11 patients (63.6%) achieving a major improvement after 6 months of treatment. Notably, High level of baseline serum IL-6 levels is one of the potential predictors of response to tocilizumab therapy (12). Beyond its efficacy in generalized myositis, tocilizumab has also demonstrated therapeutic benefit in orbital myositis. Zhang et al. reported a patient with myositis-type idiopathic orbital inflammation accompanied by extra-ocular muscle thickening, diplopia, and restricted ocular movement, who achieved marked clinical and radiographic improvement after four monthly infusions of tocilizumab (8 mg/kg), with resolution of diplopia and reduction of extra-ocular muscle swelling on MRI (13). In our case, serum IL-6 levels were elevated both at disease onset and prior to tocilizumab initiation. Following tocilizumab treatment, the patient experienced significant improvement in binocular diplopia and ocular motility. This observation further supports the notion that tocilizumab is effective in refractory myositis involving the extra-ocular muscles. Moreover, measuring serum IL-6 levels before tocilizumab therapy may help predict treatment response in such patients.

The differential diagnosis in this case included other forms of myositis such as focal myositis, proliferative myositis, ischemic myositis, and granulomatous myositis. Focal myositis typically remains confined to a single muscle group and lacks systemic inflammatory markers; the presence of multifocal lesions, elevated ESR and CRP, and eosinophilic infiltration in our patient argued against this diagnosis (14). Proliferative myositis, characterized by excessive fibroblastic proliferation, was unlikely given the involvement of skeletal muscle fascicles. Ischemic myositis, often seen in elderly or debilitated patients, was excluded based on the patient’s clinical profile and histopathological findings. Granulomatous myositis was ruled out by the absence of epithelioid granulomas or multinucleated giant cells on biopsy (15).

This case has several limitations inherent to a single case report. The therapeutic response to tocilizumab was observed in combination with ciclosporin and low-dose prednisone, so the precise contribution of each agent cannot be definitively determined. Additionally, the long-term durability of the response and the safety profile of this combination in this specific population remain to be established. Nevertheless, the temporal association between tocilizumab initiation and clinical improvement, coupled with the failure of multiple prior therapies, strongly supports a therapeutic effect.

## Conclusion

This case represents a rare description of extra-ocular muscle involvement in focal eosinophilic myositis and the first report of successful treatment with tocilizumab in this context. The unusual ocular manifestation was associated with a refractory disease course, suggesting that extra-ocular muscle involvement may define a more treatment-resistant phenotype. Tocilizumab, an IL-6 receptor antagonist, emerged as an effective therapeutic option when conventional immunosuppressive therapies failed. Further studies, including additional case reports and case series, are needed to validate these findings and to elucidate the mechanisms underlying the distinctive clinical behavior of FEM with extra-ocular involvement.

## Data Availability

The original contributions presented in the study are included in the article/supplementary material. Further inquiries can be directed to the corresponding author.
